# Characterizing Online Opinion on Vaping and E-Cigarettes in X (Formerly Twitter) Discourse in Singapore: Infodemiology Study

**DOI:** 10.2196/103561

**Published:** 2026-09-24

**Authors:** Charles Alba

**Affiliations:** 1 Division of Computational and Data Sciences, Washington University in St. Louis, 1 Brookings Drive, St. Louis, MO, 63130, United States, 1 314-935-5000

**Keywords:** e-cigarettes, vaping, Singapore, infodemiology, social media mining

## Abstract

As Singapore enforces a comprehensive e-cigarette prohibition, this infodemiology analysis of vaping-related X (formerly Twitter) discourse reveals predominantly neutral, event-driven discussions across 18 topics covering legislation and policy, illicit consumption, illicit sales and smuggling, and health risks, offering insights to inform vaping control strategies.

## Introduction

E-cigarettes remain a contested public health issue, with countries adopting divergent regulatory approaches. Singapore has maintained a comprehensive prohibition on the importation, sale, distribution, and possession of e-cigarettes since 2018, with recently strengthened enforcement measures and penalties [1-4]. While understanding public response to these measures is important, survey- and interview-based approaches are subject to bias from disclosure fears around legal repercussions [1], making social media a valuable complementary data source. This study aims to leverage text-mining tools to provide a fine-grained characterization of vaping-related discourse on social media, with the goal of informing potential strategies for vaping policies.

## Methods

### Ethical Considerations

Washington University’s institutional review board deemed this study to be exempt from review. All data used for this study have been deidentified.

### Data

In total, 4541 posts from X were collected using X’s PRO API through a curated set of vaping-related keywords and hashtags (detailed in Multimedia Appendix 1), spanning January 1, 2017—a year before the prohibition on the purchase, possession, and use of e-cigarettes on February 1, 2018—through September 20, 2025—the date of data collection.

### Analysis

Sentiment analysis was first used to classify X posts as positive, negative, or neutral. This was followed by applying topic modeling, a text-mining technique that clusters contextually similar posts into keyword-represented topics, independently. Topics were then analyzed across sentiments over time.

For sentiment analysis, Tabularis AI’s multilingual sentiment analysis model from Hugging Face [5] was used because of its multilingual capabilities, allowing it to classify sentiments covering Singapore’s 4 national languages. This is detailed in Multimedia Appendix 1.

For topic modeling, BERTopic [6] was used to uncover themes in vaping-related discourse. As a clustering-based topic modeling technique, BERTopic does not require predefined labels; instead, it naturally groups X posts into topics based on similarities in their semantic meanings.

Parameters are detailed in Multimedia Appendix 1.

## Results

**Figure 1.**
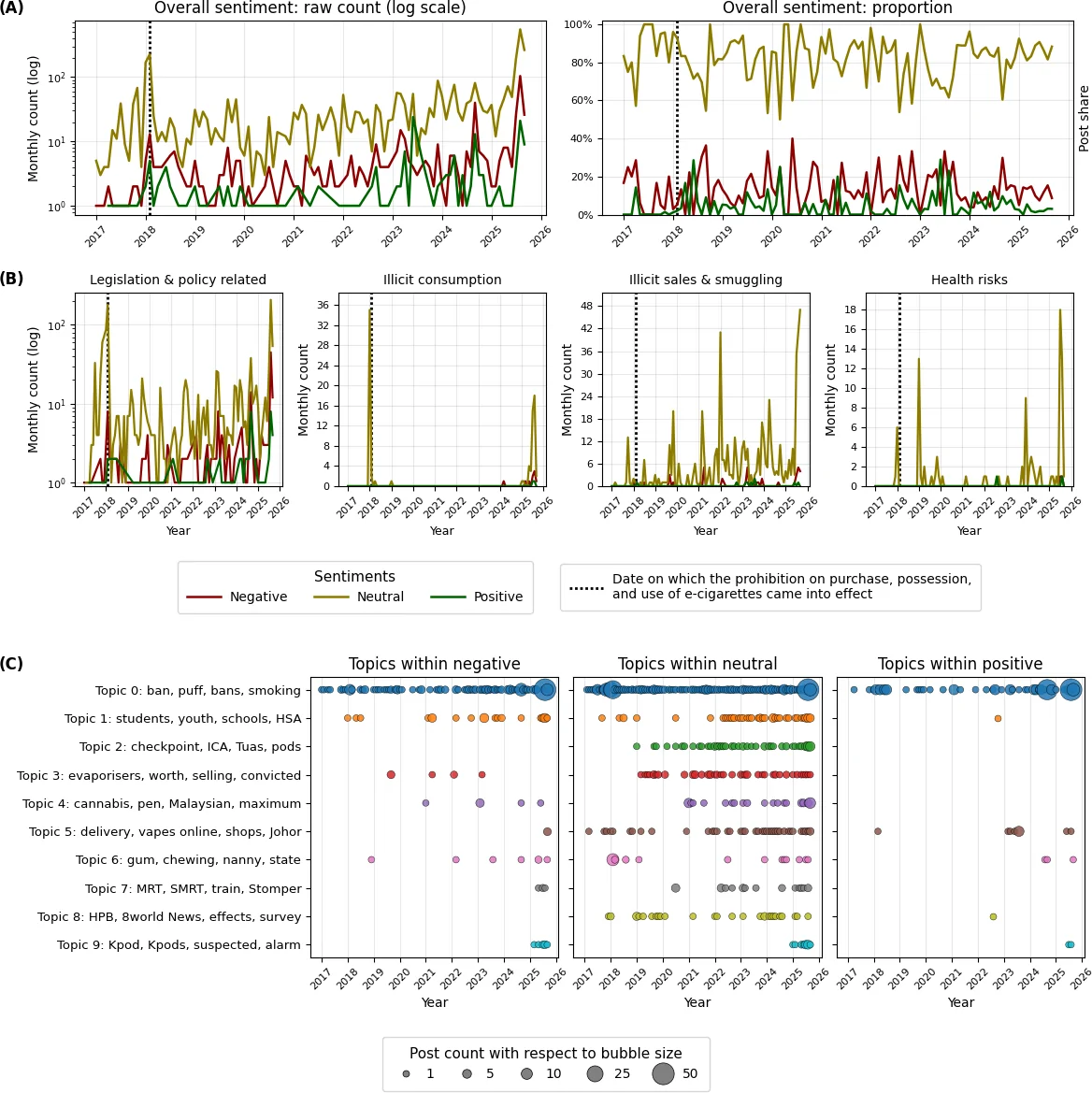
Sentiments of vaping-related X posts (formerly tweets) from Singapore spanning January 1, 2017, to September 20, 2025. (A) Temporal trends of sentiments spanning the entire corpus; (B) sentiments by post category; and (C) breakdown of sentiments across the 10 most frequent topics over time. HPB: Health Promotion Board; HSA: Health Science Authority; ICA: Immigration & Checkpoints Authority; MRT: Mass Rapid Transit; SMRT: Singapore Mass Rapid Transit.

Descriptive statistics are detailed in Table S1 in Multimedia Appendix 2. Figure 1 illustrates the sentiment and topic composition across time, showing that discourse is predominantly neutral, followed by negative and then positive. Nonetheless, there were occasional periods where positive sentiment exceeded negative, notably around mid-2018, mid-2023, and mid-2024.

Table 1 reveals 18 topics identified through BERTopic, which can be grouped into 4 dimensions: (1) legislation and policy-related, including vape ban legislation (topics 0, 10) and nanny state critiques akin to gum-related laws (topic 6); (2) illicit consumption, covering enforcement-related incidents involving students, train and cable car commuters, and government officials (topics 1, 7, 13, 15); (3) illicit sales and smuggling, including smuggling via borders (topics 2, 4, 16), black market sales (topics 5, 17), and distribution via messaging platforms (topic 11); and (4) health risks and substances.

**Table 1.** Topics identified through BERTopic analysis of X posts sorted by topic frequency. Each topic is represented by keywords and grouped into 4 categories, with representative text(s) from each topic provided.

Topic	Count	Topic keywords	Interpretation	Examples of quoted posts	Category
0	1432	Ban, puff, bans, smoking	Ban on vapes	“This is what war on vaping looks like. Time will tell if criminalizing people for making safer choices will lead to lower tobacco deaths.”; “Vaping is banned in Singapore — and for good reason.... Courtesy means caring for health 🦁*”*	Legislation & policy-related
1	167	Students, youth, schools, HSA^a^	Vaping among youths and students	“Vaping culture is just another example of how student residents are failing to abide by institutional rules and regulations - it’s no surprise given the current lax attitudes of young adults....”	Illicit consumption
2	160	Checkpoint, ICA^b^, Tuas, pods	Smuggling vapes in border checkpoints	“Not cool, ‘ma... 🥺 On Christmas Eve, ICA officers foiled an attempt to smuggle e-vaporisers and accessories via a Singapore-registered car driven by a female Singaporean....*”*	Illicit sales & smuggling
3	127	Evaporiser, worth, selling, convicted	Individuals convicted for illicit e-cigarette sale	“14 charged with selling e-vaporisers and other items, fined $255,500*”*	Illicit sales & smuggling
4	113	Cannabis, pen, Malaysian, maximum	Smuggling cannabis pens from Malaysia	“300K illicit vapes seized in Malaysia, destined for Singapore’s black market. Smuggling like this fuels organized crime and tax losses. Stronger enforcement and cross-border cooperation are key....*”*	Illicit sales & smuggling
5	83	Delivery, vapes online, shops, Johor	Black market for vapes in Johor and online	“This video says it all. Vaping is prevalent in Singapore & locals as well as couriers are willing to risk it all just to feed their habits or sell at a profit. Johor Bahru vape shops say Singaporeans still form the bulk of their customers*”*	Illicit sales & smuggling
6	78	Gum, chewing, nanny, state^c^	Nanny state criticism of vaping legislation akin to gum laws	“Singapore authorities will hang you for chewing gum and vaping but welcome thousands of notorious financial criminals to party on their 10 acre plot of land... Crazy.*”*	Legislation & policy-related
7	68	MRT^d^, SMRT^e^, train, Stomper	Individuals caught vaping in trains	“A teen was filmed stumbling on an MRT train; SMRT staff observed him allegedly vaping and handed him over to authorities..... 👍🏽 👌🏽🙏🏽 lemak! Bodohhh [fat! Stupid]*”*	Illicit consumption
8	61	HPB^f^, 8world News, effects, survey, dangers	Dangers and effects of vaping	“The national prevalence of the use of e-cigarettes and other vaping products jumped to 14.9 per cent in 2022.”; “7 in 10 youth are unaware of e-cigarette dangers*”*	Health risks & substances
9	54	Kpod, kpods, suspected, alarm	Alarming Kpod use	“I wonder: are KPODS an actual thing? A quick google search seems to reveal no matches outside of Singapore. However inside Singapore there is a supposed KPOD crisis which are drug laced vapes that turn you into a zombie, And this triggers harsher penalties for vape users.”	Health risks & substances
10	40	Amended, act, tobacco, illegal, e-cigarettes	E-cigarettes ban during the amended Tobacco Act	“E-cigarettes, shisha will be illegal from Feb 1 under amended Tobacco Act....*”*	Legislation & policy-related
11	38	Hawking, network, contraband, Telegram	Illicit sale networks via Telegram	“Learn out. Singapore busts network hawking contraband e-vaporisers via Telegram*”*	Illicit sales & smuggling
12	35	Etomidate, drug, anesthesia, MDA^g^	Classification of etomidate as a Class C drug	“Singapore is moving to classify etomidate, an anesthetic found in vapes, as a Class C drug under the Misuse of Drugs Act (MDA*).”*	Legislation & policy-related
13	27	Cable, Sentosa, teens, car, lodge	Individuals caught vaping in Sentosa cable cars	“Back in my day, when teens want to do stupid things, we don’t boast or show off about it.... Teens film themselves... vaping in Sentosa cable car, police report lodged*”*	Illicit consumption
14	26	Criminals, prison, vapers, smugglers	Prison sentence for vape sellers and smugglers	“Singapore: Four Serve Prison Terms for Selling Vapes.*”*	Illicit sales & smuggling
15	19	NEA^h^, summons, dismissed, illegally	Government officials caught illegally vaping	“Broke the hes supposed to control... NEA officer caught vaping after issuing summons.*”*	Illicit consumption
16	13	177, Changi^i^, airport, operation	Airport operations in curbing e-cigarette smuggling	“177 people arriving at Changi Airport found with vapes in multi-day operation”	Illicit sales & smuggling
17	12	Booming, market, ecigarette, rising	Booming e-cigarette black market	“E-Cigarette black market booming in Singapore: Importing and selling vaping products have been...”	Illicit sales & smuggling
-1	1988	*Outlier*	*Outlier*	*Outlier*	—^j^

a HSA: Health Science Authority.

b ICA: Immigration & Checkpoints Authority; agency responsible for border control in Singapore ports.

c Chewing gum is banned in Singapore.

d MRT: Mass Rapid Transit; rapid transit system of Singapore.

e SMRT: Singapore Mass Rapid Transit; public transport operator in Singapore.

f HPB: Health Promotion Board; Singapore’s statutory board for national health promotion and disease prevention programs.

g MDA: Misuse of Drugs Act.

h NEA: National Environmental Agency; agency tasked with sustaining a clean environment.

i Changi Airport is Singapore’s main airport.

j Not applicable.

A breakdown of the type of account, or source of information, across topics is detailed in Multimedia Appendix 2, in addition to external evaluation of BERTopic’s identified topics and assignment of each topic to higher-level themes [7].

“Legislation and policy-related” sentiments fluctuated, with increased discourse during legislative and policy changes. Neutral-dominated discourse encompassed news- or information-sharing by individuals or news outlets on topics such as banning vapes, amendments, and etomidate’s drug classification (topics 0, 10, 12, respectively), per Multimedia Appendix 2 and the quoted examples of topics 10 and 12 in Table 1. Negative posts primarily surrounded nanny state criticisms (topic 6) and individuals opposed to blanket bans on vapes (topic 0) as demonstrated by the topic-specific quotes in Table 1. Positive posts primarily spanned support for such legislation (topic 0), although they were less pronounced than other sentiments.

“Illicit consumption” discourse increased during periods coinciding with vaping incidents involving students, train and cable car commuters, and government officials (topics 1, 7, 13, 15, respectively), including spikes in 2018 and late 2025 during heightened crackdowns. Posts were primarily neutral, encompassing information-sharing from news outlets or individuals, followed by negative sentiments, which encompassed frustration directed at the culprits and the reported incidents themselves, as best illustrated in topics 1 and 7 of the quotes in Table 1.

Similar trends were observed in “illicit sales and smuggling” discourse, where posts were predominantly neutral, followed by negative discourse, with few positive discussions and increased discourse during crackdowns. Discussions surrounded reports of smuggling via checkpoints, neighboring country Malaysia, and airports (topics 2, 4, 16, respectively), illicit sales networks via black markets (topics 5, 17) and Telegram (topic 11), and punishments for smuggling offenders (topics 3, 14). Per Multimedia Appendix 2, a portion of these posts stemmed from news outlets and nongovernmental groups. Most neutral discussions surrounded news sharing, commentary, and individual observations, per the quotes in topics 3, 4, and 5 of Table 1, respectively, while negative discussions leaned toward anger directed at smuggling incidents themselves, per the quote in topic 2 of Table 1.

Finally, discussions on “health risks and substances” were predominantly neutral, which included discussions on dangers and effects of vaping as well as Kpod use (topics 8, 9, respectively). Most discussions stemmed from news outlets disseminating findings and individuals commenting on vaping-related health issues, per Figure 1C and the topic-specific quotes in Table 1.

## Discussion

This study aimed to characterize vaping-related discourse on social media to inform potential implementation strategies, particularly as survey-based approaches are susceptible to disclosure bias in ban jurisdictions [1]. It found that discourse was predominantly neutral and event driven, with discussions on legislation, illicit consumption, sales and smuggling incidents, and health risks.

Echoing qualitative reports of regulation-over-ban preferences and skepticism toward anti-vaping messaging [2,3], such views (topics 0, 6) surfaced in negative posts within predominantly neutral discourse, while positive posts endorsed legislation. The illicit market and youth vaping culture documented qualitatively [2-4] proved publicly visible: neutral reporting dominated (topics 1, 5, 11), with negativity targeting offenders.

These findings also point to implementation frameworks that could guide vaping control efforts. The Consolidated Framework for Implementation Research [8] could help address public acceptability concerns such as nanny state criticisms; the dynamic sustainability framework [9] could inform adaptive enforcement as illicit market channels shift; and policy feedback theory [10] could explain the apparent mutual reinforcement between enforcement actions and public support.

Nonetheless, this study has limitations: data were drawn exclusively from X, which may not represent broader public opinion, with user demographics and location often masked.

As vaping policies evolve worldwide, infodemiology can complement conventional methods to support responsive and adaptive implementation strategies.

## Supplementary material

10.2196/103561 Multimedia Appendix 1 Additional methods: search query, further explanation of sentiment analysis, and sentiment analysis and BERTopic parameters.

10.2196/103561 Multimedia Appendix 2 Additional results: breakdown of account types across corpus, manual topic evaluation, and assignment of topics into higher level groups.
