# Supplementary material for: Characterizing Online Opinion on Vaping and E-Cigarettes in X (Formerly Twitter) Discourse in Singapore: Infodemiology Study
Source: JMIR Infodemiology. 2026 Sep 24;6:e103561. doi: 10.2196/103561 (PMC13608228; doi:10.2196/103561)
Supplement: Supplementary file 1 [file infodemiology-v6-e103561-s001.docx]

## Search Query

*(*

*vape OR vapes OR vaped OR vaping OR vaper OR vapers*

*OR "e-cig" OR "e-cigs" OR ecig OR ecigs OR "e cig"*

*OR "e-cigarette" OR "e-cigarettes"*

*OR "e-vaporiser" OR "e-vaporisers" OR "e-vaporizer" OR "e-vaporizers" OR "e-vapouriser" OR "e-vapourisers"*

*OR "nic salt" OR "nic salts"*

*OR podsystem OR "pod system" OR "pod systems" OR "pod mod" OR "pod mods"*

*OR "disposable vape" OR "disposable vapes"*

*OR juul OR relx OR "elf bar" OR elfbar OR "geek bar" OR geekbar OR hqd OR moti OR myle OR uwell OR caliburn OR oxva OR vaporesso*

*)*

*(*

*singapore OR "singapore's" OR singapores OR singaporean OR singaporeans*

*OR "s’pore" OR "s’pore’s" OR "s'pore" OR "s'pore's"*

*OR sgpore OR "sgpore's"*

*OR HSAsg OR @HSAsg OR @SingaporePolice*

*)*

*-is:retweet -is:reply*

*-(podcast OR podcasts OR airpods OR "coffee pods")*

## Further explanation of sentiment analysis

Sentiment analysis is a natural language processing technique used to identify the tone and stance of a given text, specifically by classifying data into positive, negative, or neutral categories to help gauge public opinion. The *Tabularisai/multilingual-sentiment-analysis* sentiment analysis model was used in-lieu of its multi-lingual capabilities, which also covered Singapore’s 4 national languages. *Tabularisai/multilingual-sentiment-analysis* is a trained model, meaning it has been trained to identify sentiments from massive amounts of hand-annotated and validated texts, allowing it to detect sentiments across distinct contexts and usages. For more information on the model, please refer to their model card at: <https://huggingface.co/tabularisai/multilingual-sentiment-analysis>

## Sentiment analysis and BERTopic parameters

| **Component** | **Hyperparameter** | **Value Used** |
| --- | --- | --- |
| Sentiment Analysis | *Tabularisai/multilingual-sentiment-analysis* | |
| Topic representation | MMR diversity | 0.1 |
| Topic reduction | nr_topics | 30 |
| Reproducibility | Random state | 42 |
| Handling non-English Tweets | Use LLM to translate text | *Meta-Llama-3.1-8B* |
