# Supplementary material for: Characterizing Online Opinion on Vaping and E-Cigarettes in X (Formerly Twitter) Discourse in Singapore: Infodemiology Study
Source: JMIR Infodemiology. 2026 Sep 24;6:e103561. doi: 10.2196/103561 (PMC13608228; doi:10.2196/103561)
Supplement: Supplementary file 2 [file infodemiology-v6-e103561-s002.docx]

## Summary Statistics

*Table S1. Descriptive statistics for the corpus of X posts.*

| **Statistic** | **Mean** | **Median** | **Standard Deviation** |
| --- | --- | --- | --- |
| Likes | 10.11 | 0.00 | 167.82 |
| Retweets | 4.10 | 0.00 | 89.21 |
| Replies | 0.82 | 0.00 | 4.56 |
| Word Count | 20.15 | 16.00 | 11.56 |
| Vocabulary Count | 19.15 | 16.00 | 10.31 |
| Character Count | 149.46 | 126.00 | 73.80 |

## Type of account or source of information posting across topics

Among each of the topics identified, I broke down and illustrated type of account or source of information posting within a theme. Categories included (1) Individual Citizens, (2) News/Media outlets, (3) Government/regulatory or civil society organizations, (4) Industry/commercial or others (including pro- or anti-vaping lobbying groups, non-profits or anything that does not fit into the prior 3 categories). Because identifying each type of account and source of information posting by hand at scale can be challenging, we leveraged the *claude-fable-5* to classify each given X post into one of four categories.

Figure A2.1: Breakdown of account types or source of information posting across topic identified through BERTopic

## Manual topic evaluation and assignment into higher level groups

While BERTopic is an unsupervised machine learning algorithm, meaning that labels are naturally identified based on the contextual semantic similarity of texts as opposed to being categorized into pre-defined labels, its manual and human evaluation is necessary to ensure its reliability when interpreting the results.

Resultingly, we selected a subset of random posts, identified based on the formulated threshold defined by Alba et al [reference 7 in main manuscript] for partial evaluation of AI-aided labels, and outsourced 3 anonymous annotators via the Prolific Platform to evaluate the accuracy of the topics identified by BERTopic. Each evaluator was paid $30 for their effort.

The majority verdict (at least 2 of 3) served as the final label. Inter-judge reliability was extremely high (Fleiss' κ = 0.83, 95% CI 0.67–0.98, p < .001; Gwet's AC1 = 0.96, 95% CI 0.92–1.00, p < .001). BERTopic's topic-assignment accuracy was 91.0% (95% CI 83.8–95.2%), and macro-averaged precision across the topics was 91.8% (bootstrap 95% CI 87.8–94.4%).

Similarly, we tasked these same evaluators to assign each topic into higher level groups. Specifically, for each topic, we asked the annotators to group each topic into one of 4 categories: (1) Legislation & Policy-related, (2) Illicit consumption, (3) Health Risks and Substances, (4) Illicit sales & smuggling. The majority verdict (at least 2 of 3) served as the final category label as well. Inter-judge reliability was extremely high (Fleiss' κ = 0.95, 95% CI 0.84–1.00, p < .001; Gwet's AC1 = 0.95, 95% CI 0.85–1.00, p < 0.001).
